# Fibrillization of 40-residue β-Amyloid Peptides in Membrane-Like Environments Leads to Different Fibril Structures and Reduced Molecular Polymorphisms

**DOI:** 10.3390/biom10060881

**Published:** 2020-06-08

**Authors:** Qinghui Cheng, Zhi-Wen Hu, Yuto Tobin-Miyaji, Amy E. Perkins, Terrence Deak, Wei Qiang

**Affiliations:** 1 Department of Chemistry, State University of New York at Binghamton, Binghamton, NY 13902, USA; qcheng2@binghamton.edu (Q.C.); zhiwenhu@binghamton.edu (Z.-W.H.); ytobinm1@binghamton.edu (Y.T.-M.); 2 Department of Psychology, State University of New York at Binghamton, Binghamton, NY 13902, USA; aperkins@binghamton.edu (A.E.P.); tdeak@binghamton.edu (T.D.)

**Keywords:** β-amyloid fibrils, biological membranes, structural polymorphisms, synaptic plasma membranes

## Abstract

The molecular-level polymorphism in β-Amyloid (Aβ) fibrils have recently been considered as a pathologically relevant factor in Alzheimer’s disease (AD). Studies showed that the structural deviations in human-brain-seeded Aβ fibrils potentially correlated with the clinical histories of AD patients. For the 40-residue Aβ (Aβ_40_) fibrils derived from human brain tissues, a predominant molecular structure was proposed based on solid-state nuclear magnetic resonance (ssNMR) spectroscopy. However, previous studies have shown that the molecular structures of Aβ_40_ fibrils were sensitive to their growth conditions in aqueous environments. We show in this work that biological membranes and their phospholipid bilayer mimics serve as environmental factors to reduce the structural heterogeneity in Aβ_40_ fibrils. Fibrillization in the presence of membranes leads to fibril structures that are significantly different to the Aβ_40_ fibrils grown in aqueous solutions. Fibrils grown from multiple types of membranes, including the biological membranes extracted from the rats’ synaptosomes, shared similar ssNMR spectral features. Our studies emphasize the biological relevance of membranes in Aβ_40_ fibril structures and fibrillization processes.

## 1. Introduction

The deposition of fibrillar β-Amyloid (Aβ) aggregates around neuronal cells is pathologically significant in Alzheimer’s disease (AD) [1,2,3]. Molecular polymorphisms in Aβ fibrils have long been discovered through the characterizations of their molecular structures, which are known to be sensitive to the growth conditions of fibrils in aqueous environments [4,5]. The pathological significance of structural polymorphisms in Aβ fibrils has recently been unraveled. Importantly, solid-state Nuclear Magnetic Resonance (ssNMR) spectroscopy studies on the molecular structures and structural deviations of the human-brain-seeded Aβ fibrils showed the potential correlation between the structural polymorphism and the clinical histories of AD patients [6,7]. Particularly for the 40-residue Aβ (Aβ_40_) fibrils, there was likely to be a predominant molecular structure in patients with distinct clinic symptoms [7,8]. Given the fact that the environmental conditions for fibrillization in these patients should be highly diverse, it is important to ask what biological factors may reduce the structural heterogeneity in vivo.

The enzymatic processing of amyloid precursor protein (APP) that produces Aβ peptides occurs in the interiors of membrane bilayers [9,10]. It was found that Aβ concentrated in certain membrane domains such as the lipid rafts [11]. Therefore, membranes may serve as a biologically relevant environment in the aggregation process of Aβ peptides. A number of studies, including our previous works, demonstrated that Aβ fibrillization-induced different types of membrane disruption in systems from model phospholipid bilayers to synaptic plasma membranes in living cells [12,13,14,15,16,17,18,19,20,21]. On the other hand, the presence of membranes also influences Aβ’s fibrillization processes as well as the structures of resultant fibrils. The lag period in thioflavin-T (ThT) fluorescence kinetics assay, which was commonly used to quantify the fibrillization rates, became longer (and, therefore, slower fibrillization rates) when Aβ aggregated in the presence of neutral phospholipid bilayers compared to nonmembrane scenarios [22]. On the contrary, the fibrillization became more rapid when there were more abundant negatively charged phospholipids in membranes [23]. In addition, fibrillization was decelerated at higher lipid to peptide molar ratios [18]. Although high-resolution structural characterizations on Aβ fibrils have been performed extensively in aqueous growth conditions, there have been much fewer works on the fibril structures in the presence of membranes. One work involving model phosphatidylcholine (PC)/phosphatidylglycerol (PG) bilayers showed that the backbone second structure of Aβ_40_ fibrils changed significantly [24]. Molecular-level structural polymorphisms have been demonstrated for the fibrils grown in aqueous solutions. It is prompt to determine whether membrane-like environments will have influences on the structural polymorphisms of fibrils.

## 2. Materials and Methods

### 2.1. Peptide Synthesis and Purification

All Aβ_40_ peptides, including the unlabeled and selectively isotope-labeled ones for ssNMR measurements, were synthesized manually by routine solid-phase peptide synthesis protocols with FMOC Chemistry. The crude peptides were cleaved using a mixture of trifluoroacetic/phenol/water/1,2-ethanedithiol/thioanisole with the volume ratio 90:5:10:5:2.5. All peptides were purified on a High-performance Liquid Chromatography (HPLC 1200 Series, Agilent Inc., Santa Clara, CA, USA.) installed with C18 reversed-phase column, and lyophilized and stored at –20 °C freezers before usage. The purified peptides were verified with LC-MS/ESI (LCMS-2020, SHIMADZU Inc., Torrance, CA, USA.) to confirm >95% purity.

### 2.2. Liposome Preparation for Model Bilayers

All phospholipids [1,2-dimyristoyl-sn-glycero-3-phosphocholine (DMPC), 1,2-Dipalmitoyl-sn-glycero-3-phosphoethanolamine (DPPE), 1,2-dimyristoyl-sn-glycero-3-phosphoglycerol (DMPG)], cholesterol, sphingomyelin (Brain, Porcine), and ganglioside GM1(Brain, Ovine-Sodium Salt) were purchased from Avanti Polar Lipids, Inc. The triglycerides were purchased from Sigma Aldrich Inc. To prepare liposomes, the combination of lipids, cholesterol, and/or triglycerides with the designed molar ratio (see Table 1 for detailed molar ratios) were mixed together in chloroform. Chloroform was removed using N_2_ flow, and the lipid films were extensively dried under vacuum overnight. The lipid film was then resuspended in HEPES buffer (4 mM, pH 7.4, 0.01% NaN_3_), followed by 10 freeze–thaw cycles and extrusion through 200 nm (for non-TG vesicles) or 1000 nm (for TG-containing vesicles) pore size membrane for 30 cycles.

### 2.3. Liposome Preparation from Isolated Synaptic Plasma Membranes from Rats

Adult male Fischer 344 rats (*n* = 6–10 per group) were procured at 3-, 12-, and 18-month-old at arrival, corresponding to young, middle-aged, and late aging, respectively. All animals were obtained from the National Institute of Aging (NIA) colony maintained by Charles River Laboratories. Subjects were given at least 2 weeks to acclimate to the colony prior to experimentation. Colony conditions were maintained at 22 ± 1 °C with a 12:12 light/dark cycle (lights on 0700). All rats were pair-housed and provided ad libitum access to food and water. On each of 2 d prior to tissue harvest, rats were handled for 2–3 min, during which time rats were weighed and marked with a nontoxic permanent marker for identification purposes. At all times, rats were maintained and treated in accordance with the guidelines set forth by the Institute of Laboratory Rat Resources (1996) and in accordance with the protocol approved by the IACUC at Binghamton University.

Rats were killed by rapid decapitation and regions of interest were dissected using microforceps and separated bilaterally, including the hippocampus and cortex. Tissue was flash-frozen in isopentane on dry ice and stored at −80 °C until use.

Literature protocols were followed for the extraction of rats’ synaptic plasma membrane vesicles (SPMVs) [25]. Briefly, the rats’ brain tissue was homogenized using a glass homogenizer with 12 strokes over a 30 s time period in 0.32 M HEPES-buffered sucrose solution (4 mM HEPES, pH 7.4). The solution was then centrifuged at 900× *g* for 10 min at 4 °C, and the pellet was collected and resuspended in 0.32 M HEPES-buffered sucrose. The solution was centrifuged for the second time at 10,000× *g* for 15 min at 4 °C and the pellet was collected again. The pellet was lysed in 4 mL deionized water and transferred to a glass homogenizer and then homogenized with 3 strokes. Then 16 uL of 1.0 HEPES buffer was quickly added to the above solution to reach the final HEPES concentration 4.0 mM. This diluted solution was then rotated at 4 °C for 30 min for a completed lysing and was centrifuged at 25,000× *g* for 20 min at 4 °C. The pellet was collected, from where the SPMs were enriched in a discontinuous sucrose gradient containing 1.2 M, 1.0 M, and 0.8 M HEPES-buffered sucrose solution layers with the volume ratio 3.5:3.0:3.0. The SPMVs were collected by centrifuging the depositions with sucrose gradient solutions at 150,000× *g* for 2 h at 4 °C, and the sample (at the bottom of the 1.0 M / 1.2 M HEPES-buffered sucrose solution interphase) was collected using an 18 G needle and a 1 mL syringe. The pellet was resuspended finally in a 4.0 mM HEPES buffer and stored at −80 °C until use.

### 2.4. Quantification of Total ^31^P in SPMs

We determined in previous works that the molar ratio between Aβ_40_ and total lipids influenced the peptide aggregation pathways significantly [18,20]. In the present study, we utilized ^31^P NMR spectroscopy to semi-quantitatively analyze the total lipids in SPMVs. Literature protocols to extract the lipids from SPMs were followed [26]. Briefly, the enriched SPMVs were dissolved in a mixture of methanol and chloroform (2:1 *v/v*) and vortexed vigorously for 1 min and rotated at ambient temperature for 30 min. The mixture was centrifuged for 5 min at 600× *g* and 4 °C on a benchtop centrifuge and the supernatant was collected. The supernatant was dried with N_2_ flow followed overnight under vacuum, and the resulting lipid film was re-dissolved in CDCl_3_ for ^31^P NMR spectroscopy with direct excitation pulse sequence. The standard curve with DMPC was obtained using the same protocol for quantification. The total lipid concentrations in 3-month, 12-month, and 18-month rats’ SPMs were quantified as 530.5 ± 2.2 µM, 755.2 ± 0.4 µM, and 777.9 ± 0.2 µM, respectively.

### 2.5. Lipid Analysis by Thin-Layer Chromatography (TLC)

**Figure 1:**
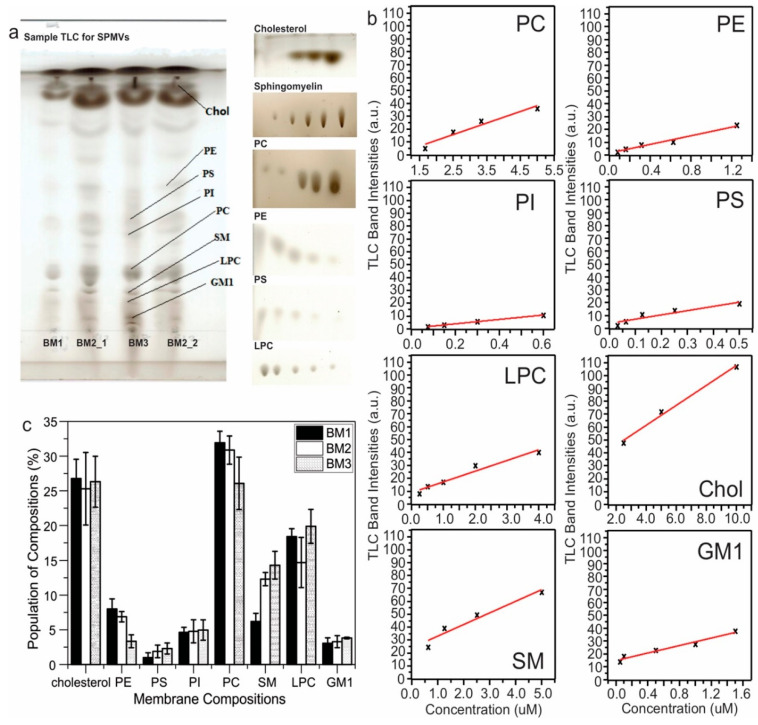
(**a**) Representative Thin-Layer Chromatography (TLC) analysis of the membrane compositions of rats’ synaptic plasma membrane vesicles (rat synaptic plasma membrane vesicles (rSPMV), left side) and TLC for the standard lipids/cholesterol at different concentrations. (**b**) Standard curves for the quantifications of membrane compositions using TLC. (**c**) Plots of lipid compositions in rSPMV of different ages. Error bars were generated from three independent TLC analyses.

Major lipid components in SPMVs of different ages were analyzed using TLC with published protocols [27]. Lipids and cholesterol compositions were extracted from SPMs as described in the previous section. The lipids were then dissolved in chloroform/methanol mixture (1:2, *v/v*) and loaded dropwise onto 20 × 10 cm TLC silica gel glass plates. Each sample loading normally contains a 2.0 µL lipid solution. Three developing solutions were applied: Firstly with a mixture of ethyl acetate/1-propanol/chloroform/methanol/potassium chloride in 12.5:12.5:12.5:6.5:4.5 volume ratio; secondly with a mixture of n-hexane/diethyl ether and acetic acid in 70:30:1 volume ratio; and finally with n-hexane. The TLC plates were then stained with 10% copper sulfate containing 9% phosphoric acid and baked at 150 °C for 10 min. The resulting plates were scanned and images (sample image shown in Figure 1) were analyzed using ImageJ software to obtain information about the intensities of individual bands.

To obtain a quantitative analysis of each of the essential lipid compositions, standard curves were obtained for eight different types of lipids/cholesterol (DMPC, DPPE, 1,2-Dipalmitoyl-sn-glycero-3-phosphoserine, sodium salt (DPPS), 1,2-dioleoyl-sn-glycero-3-phospho-(1’-myo-inositol) (ammonium salt, DOPI), 1-palmitoyl-2-hydroxy-sn-glycero-3-phosphocholine (LPC) ], sphingomyelin (Brain, Porcine) and ganglioside GM1(Brain, Ovine-Sodium Salt)), which represented the main assigned lipid compositions from SPMVs in the literature [27]. TLC with standards at different concentrations was obtained using the same protocol as for the SPMV samples, and the standard curves (Figure 1) were obtained by plotting the band intensities as a function of concentrations. The absolute band intensities from ImageJ for the individually assigned lipid compositions in SPMVs were converted to concentrations based on the standard curves, and the population percentages for individual components were plotted in Figure 1.

### 2.6. Thioflavin-T (ThT) Fluorescence Kinetics on Fibrillation

ThT fluorescence assays were performed on Synergy™ HTX Multi-Mode Microplate Reader (BioTek Instruments, Inc., Winooski, VT, USA) with excitation and emission wavelengths at 450 and 480 nm, respectively. A 150 μL volume of each one of the freshly prepared samples (Aβ_40_ peptide concentration ~10 μM and peptide-to-membrane ratio 1:30, buffer-4 mM HEPES with pH 7.4 and 0.01% NaN_3_) was pipetted to the 96-well plate and the concentration of the ThT solution (dissolved in 4 mM HEPEs buffer pH 7.4, 0.01% NaN_3_) was controlled to be 50 μM. The kinetic measurements (1 data point per hour) were performed at 37 °C and 10 s shaking at a rate of 307 rpm was applied in order to make sample solutions as homogeneous as possible before measurements. All samples were analyzed triplicate with controls that contained only vesicles but not the additions of Aβ_40_ peptides.

To analyze the ThT kinetics curves, the traces from samples were, firstly, subtracted from their corresponding controls, and then fitting to the sigmoidal growth function:

(1) $$Intensity=y0+A0/\left(1+\exp\left(-k\left(t-t_{\frac{1}{2}}\right)\right)\right),$$

where y0 is the baseline intensity, A0 is the amplitude of intensity changes, *k* is the apparent slope, and t_1/2_ is the half-maximum time. The lag period (t_lag_) was calculated as t_1/2_ – 2/*k*. The maximum fluorescence intensity, the lag period, and the growth rate constant were reported in Table 2. Error bars were calculated at the standard deviations from three independent measurements for each model membrane.

### 2.7. Membrane Content Leakage Assay

The calcein fluorescence assay was applied as an indicator for phospholipid vesicle leakage in this paper and the detailed protocol was described previously [21]. Briefly, the dried lipid films (dried by nitrogen gas flow and followed under vacuum overnight) were resuspended in 10 mM sodium phosphate (pH 7.40 and 0.01% NaN_3_) -buffered 10 mM calcein solution. Then, the above vesicle solutions were subjected to 8-time freeze–thaw cycles (60 °C water bath as warming source and liquid nitrogen as cooling source) and extruded by a 200 nm membrane filter for 15 times. The pretreated vesicle solutions were loaded to snake bags with a 3.5 kDa molecular weight cutoff and dialyzed against the bulk sodium phosphate solution (1:1000) for 4 d to remove the uncaptured calcein molecules as much as possible. After dialysis, the calcein-containing liposomes were mixed with monomeric Aβ40 (final concentration was controlled to 25 µM) and immediately were pipetted to a 96-well plate. The kinetic traces were collected on a BioTek Synergy multi-mode plate reader (excitation filter ~460 ± 40 nm and emission filter ~560 ± 40 nm) at 37 °C and the data was collected every 30 min. Until the curves reached a plateau, 1% Triton-X (Sigma-Aldrich, Inc., St. Louis, MO, USA) was added to each well and final fluorescence intensity was measured.

After measurements, the data was processed by subtracting the corresponding controls (without the addition of Aβ40) and the lag phase and buildup rate were obtained by fitting to a sigmoidal curve. The percentage of membrane leakage was calculated by the following equation:

(2) $$membrane\;leakage\%=\frac{I_{t}-I_{0}\left(sample\right)}{I_{f}\left(triton\right)-I_{0}\left(control\right)}$$

where *I*_0_ and *I_t_* are the final fluorescence intensity (the plateau) and the fluorescence intensity after treated by 1% Triton-X.

### 2.8. Solid-State NMR (ssNMR) Spectroscopy

All samples for ssNMR measurements were incubated quiescently for over 2 weeks before experiments. Aβ_40_-SPMVs solutions were centrifuged (85,000 rpm, 4 °C, 1 h), lyophilized, packed into 2.5 mm magic-angle spinning (MAS) rotors, and rehydrated with deionized water (~1.0 µL/mg sample). All ssNMR experiments were performed on a 600 MHz Bruker spectrometer equipped with a 2.5 mm TriGamma MAS probe. The MAS spinning frequency was kept at 10,000 ± 2 Hz. Sample temperature was controlled at ~280 K throughout the experiments by monitoring the ^1^H chemical shift in H_2_O. All two-dimensional (2D) NMR spectra were collected with the ^13^C–^13^C spin diffusion spectroscopy with either 20 ms (for detecting intra-residue cross peaks) or 500 ms (for inter-residue cross peaks) mixing period. All spectra shown in figures were processed with 100 Hz Gaussian line broadening in both dimensions. All samples were kept at 280 ± 5 K with cooling N_2_ air and the sample temperatures were monitored before and after measurements by assessing the ^1^H chemical shifts for H_2_O molecules.

### 2.9. Negatively Stained Transmission Electron Microscopy (TEM)

TEM images were recorded on a JOEL J-2100 Electron Microscope with an 80 kV acceleration electron field. Samples were prepared by, firstly, deposited a 20 mL fibril solution on copper coded TEM grid for 2 min. The solutions were blotted and stained with 2% uranyl acetate for 30 s. The staining solution was then blotted, and the grid was rinsed with deionized H_2_O twice and dried in the air.

### 2.10. Circular Dichroism (CD) Spectroscopy

CD spectra were collected on a JASCO J-810 Spectrophotometer. Fibril solutions with different incubation times were analyzed by CD to collect the total peptide signal (top row in Figure 9) and then centrifuged (Beckmann Coulter Benchtop ultracentrifuge) for 30 min at 50,000 rpm and 4 °C to separate the membranes from the aqueous buffer (and, therefore, membrane-bound and free Aβ_40_). The supernatants were analyzed by CD to collect the free peptide signal (bottom row in Figure 9). All spectra were recorded at r.t. from 190 nm to 260 nm with 30 scans of signal averaging.

## 3. Results

### 3.1. Fibrillization of Aβ_40_ Peptides in Different Membranes Possess Distinct Kinetics

**Figure 2:**
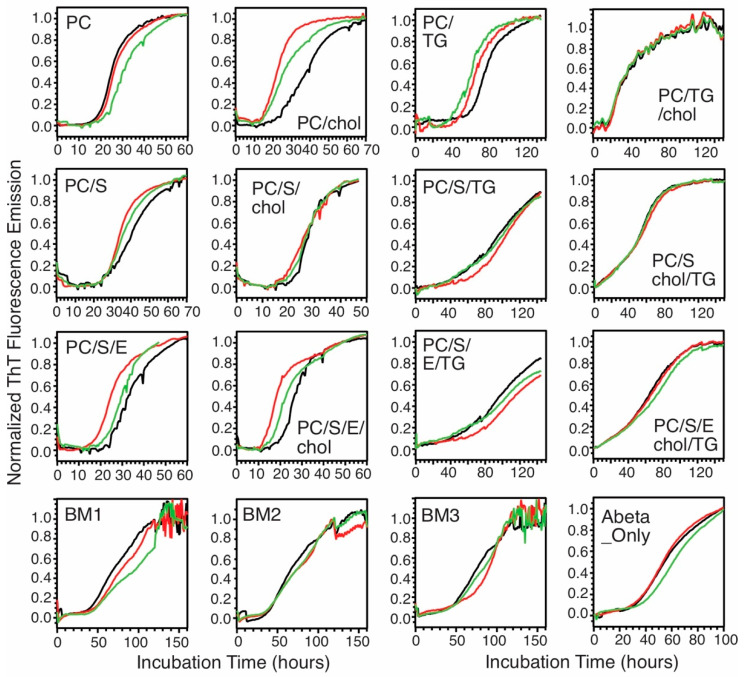
Thioflavin-T fluorescence traces (three repetitions for each membrane composition) for Aβ_40_ fibrillization kinetics in the presence of different membrane models and a control that only contains the peptides.

**Figure 3:**
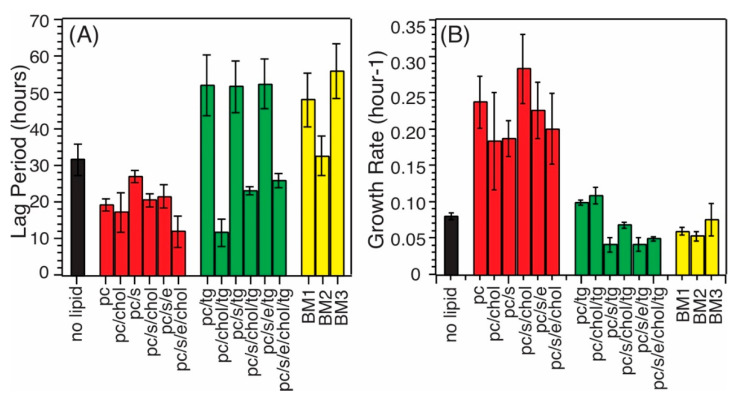
Plot of the lag periods (t_lag_, **A**) and growth rate constants (k, **B**) for Aβ_40_ fibrillization in different membrane models. Error bars were generated from three repetitions of thioflavin-T (ThT) kinetic curves shown in Figure 2. Different colors are used to facilitate the presentation (different model membranes).

Biological membranes contain multiple compositions, which naturally provide heterogeneous environments for Aβ fibrillization. This work uses Aβ_40_ peptides because the fibril structures of this particular alloform extracted from patients’ brain tissues have been shown to correlate with the clinical symptoms of AD patients [7]. We, firstly, ask whether and how the fibrillization kinetics would be influenced by different membrane compositions. We utilized in this work the biological rats’ synaptic plasma membrane vesicles (rSPMV) with different ages at 3 (BM1), 12 (BM2), and 18 (BM3) months, as well as multiple types of phospholipid bilayers with key compositions identified from the analysis of the rSPMV. The rSPMV were extracted using literature protocols [25] and the populations of essential components such as lipids and cholesterol were quantified using Thin-Layer Chromatography (TLC, Figure 1). The populations of most major phospholipids, cholesterol, and gangliosides were consistent with those reported for mammalian plasma membranes and showed no age-dependency [28]. However, interestingly, the phosphatidylethanolamine (PE) population (8.0% ± 1.4% in BM1, 6.9% ± 0.8% in BM2, and 3.3% ± 0.9% in BM3) decreased significantly at the older age. Previous studies reported that PE might enhance the Aβ-fibril-dependent membrane fragmentation and increase the lateral pressure in membrane bilayers and membrane stability [14]. In addition, the population of sphingomyelin (6.2% ± 1.2% in BM1, 12.3% ± 1.0% in BM2, and 14.3% ± 1.9% in BM3) also varied with age, but in an opposite trend. There were also large abundances of mono- and triglycerides in all three rSPMV. Inspired by the lipid analysis of rSPMV, we generated a set of model phospholipid bilayers that contained different combinations of PC, PE, phosphatidylserine (PS), chol and TG to explore the effects of lipid compositions on fibrillization kinetics. This work focuses on the effects of phospholipids, cholesterol (chol), and triglycerides (TG), while we realize that other components such as sphingolipids and gangliosides are also important components in the aggregation of Aβ peptides. Particularly, the ganglioside GM1 has been shown to promote the formation of toxic Aβ fibrils through direct Aβ–GM1 interactions [29,30]. Table 1 summarizes the model bilayers that have been utilized in this work. Figure 2 shows the full sets of thioflavin-T (ThT) fluorescence kinetic traces for all tested model membranes, for which the concentrations of Aβ_40_ were kept at 10 µM. All ThT traces showed typical sigmoidal increases for nucleation-driven fibrillization processes with distinct lag periods (t_lag_) and growth rates (k), which were analyzed and plotted in Figure 3 and summarized in Table 2. The following conclusions could be drawn: (1) the presence of TG induced significantly elongated t_lag_ values for model bilayers with most phospholipids/cholesterol compositions (except for those with PC, chol, and TG, which was similar to non-TG bilayers). In fact, the phospholipid model bilayers with TG showed t_lag_ values that were comparable to those for the biological rSPMV (i.e., ~50 h), suggesting that the presence of TG might have critical contributions to the fibrillization of Aβ in real biological membranes. This observation was consistent with previous studies that showed the inhibition effects of TG on Aβ fibrillization in aqueous environments [31]. (2) The bilayer models containing cholesterol showed decreased t_lag_ values generally in comparison to their non-cholesterol analogs, meaning that cholesterol was another biologically relevant composition in membrane bilayers. The distribution of cholesterol was known to be asymmetric across the bilayers of cellular plasma membranes and the population distribution would change with aging [32,33]. Our results imply that the distribution of cholesterol across bilayers may modulate the Aβ fibrillization processes. (3) Compared to the nonmembrane Aβ fibrillization process, the presence of phospholipids and/or cholesterol shortened t_lag_ values in general. It was previously reported that the presence of negatively charged PG would accelerate the fibrillization compared to the neutral PC bilayer [23]. However, such an effect has not been observed in the presence of work with negatively charged PS, which represents a more biologically relevant lipid composition in mammalian cells. (4) The presence of phospholipids and/or cholesterol does increase the growth rates generally compared to the fibrillization in aqueous solution (Figure 3B). The growth rates were decreased in the presence of TG compared to the non-TG analogs, confirming the effects of TG to inhibit the Aβ_40_ fibrillization. The growth rates in SPMVs were similar to those in TG-contained bilayers.

### 3.2. The Presence of Membranes Leads to Different Fibril Structures and Reduces the Structural Polymorphisms in the Resulting Fibrils

**Figure 4:**
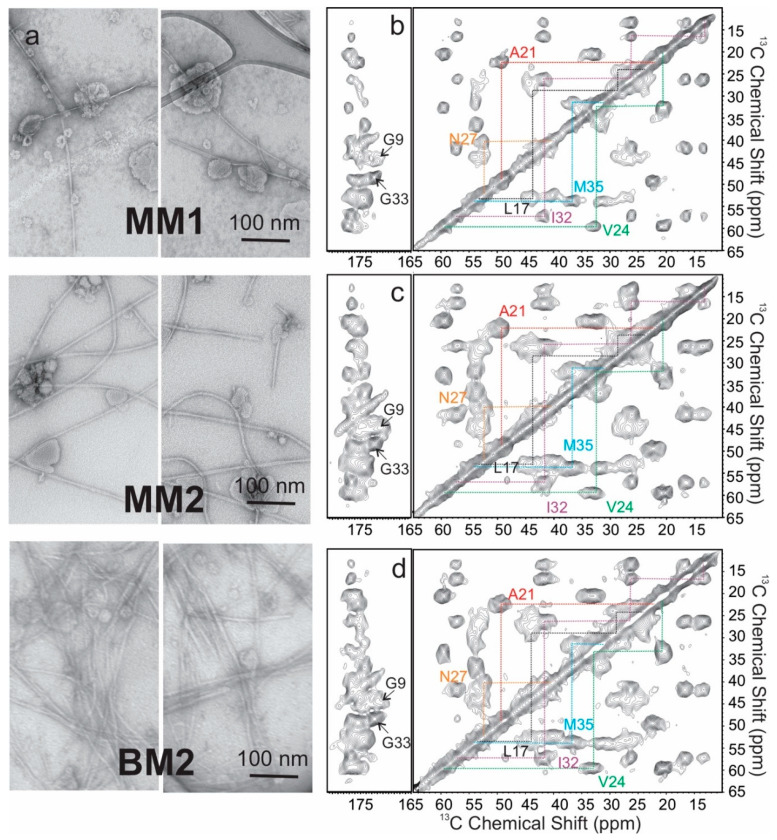
(**a**) Negatively stained transmission electron microscopy (TEM) images for Aβ_40_ fibrils grown in the presence of MM1 (top), MM2 (middle), and BM2 (bottom) model membranes (MM1: the model bilayer with 100% phosphatidylcholine; MM2: the model bilayer with phosphatidylcholine/phosphatidylglycerol/cholesterol/sphingomyelin/ganglioside GM1 at molar ratio 1.00/1.00/1.33/1.00/0.10; BM2: rat synaptic plasma membrane vesicles with 12-month age). (**b**–**d**) Representative short-mixing (20 ms) 2D ^13^C–^13^C spin diffusion spectra for Aβ_40_ fibrils formed with (**b**) MM1, (**c**) MM2, and (**d**) BM2 membrane models. The peptides were selectively uniformly labeled at residues G9, L17, A21, V24, N27, I32, G33, and M35.

Given the fact that the Aβ fibrillization kinetics was influenced significantly by membrane compositions, we next ask whether the presence of different membrane compositions would increase the structural polymorphisms in the resulting fibrils. To this end, we chose three model membrane systems with distinct *t*_lag_ values: MM1 with the simplest composition, MM2 with the most complicated non-TG compositions, and BM2 to represent the biological rSPMV. Figure 4a showed the negatively stained TEM images for the three types of Aβ_40_ fibrils, which had high similarity in terms of the bulk morphologies. Selectively isotope-labeled residues (G9, L17, F19, A21, V24, N27, I32, G33, L34, and M35) were incorporated in samples to cover a broad range of primary sequence and two typical segments of β strands in previously reported Aβ_40_ fibril structures [5]. Figure 4b–d shows the two-dimensional (2D) ^13^C–^13^C spin diffusion spectra with 20 ms mixing time for identifying the intra-residual cross peaks. All residues, except for G9 and L17, showed well-defined intra-residual cross peaks. The fact that L17 showed broadened linewidths suggested that the fibrils grown in membrane-like environments contained smaller cores compared to those grown in aqueous solutions [6,34,35,36,37,38]. The residue-specific ^13^C chemical shifts for all three fibrils were summarized in Table 3.

**Figure 5:**
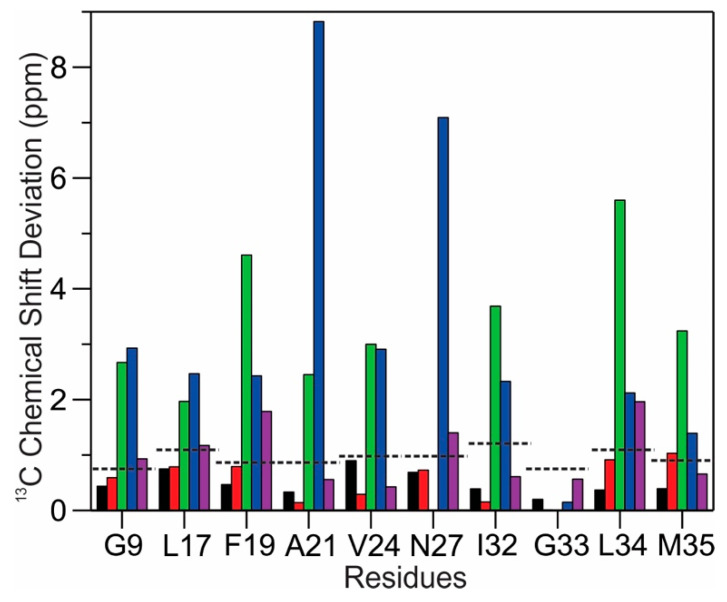
Plot of the residue-specific ^13^C chemical shift deviation for membrane-associated Aβ_40_ fibrils derived from MM1 (the fibrils grown from phosphatidylcholine (PC) model bilayers). The comparison contains two types of fibrils from current studies: those grown from the MM2 model bilayer (black) and those grown from the extracted synaptic membranes BM2 (red), and three fibrils reported in the literature: the three-fold Aβ_40_ fibrils grown from synthetic peptides (green) [35], the first molecular structure from human-brain-seeded Aβ_40_ fibril (blue) [6] and the reported predominant structure from multiple human brain tissue samples (purple) [8]. The dashed lines represent the thresholds of significant chemical shift difference for individual residues, by considering the ssNMR line widths.

We quantitatively analyzed the differences in residue-specific ^13^C chemical shifts in multiple types of Aβ_40_ fibrils, including the three membrane-associated fibril samples from the present study and three references from the literature (the three-fold Aβ_40_ fibril grown from aqueous buffer (Mref1) [35], the first brain-seeded Aβ_40_ fibril from an AD patient (Mref2) [6], and the proposed predominant Aβ_40_ fibrillar structure existed in multiple AD patients (Mref3) [8]). All these fibrils were grown from synthetic Aβ_40_ peptides under quiescent incubation at physiological temperature and pH values with similar ionic strengths. Specific types of ^13^C nuclei were considered for each individual residue, for instance for F19, we considered the ^13^C chemical shifts of C’, Ca, and Cβ, and for L34, we considered C’, Ca, Cβ, Cg, Cd1, and Cd2. The residue-specific ^13^C chemical shift deviations were calculated as

(3) $${\left[\sum_{i}{\left(\delta_{i,sample1}-\delta_{i,sample2}\right)}^{2}\right]}^{1/2}$$

where $\delta_{i,sample1}$ and $\delta_{i,sample2}$ were the ^13^C_i_ chemical shifts in two different samples. The ^13^C spectral resolution was estimated as ~0.5 ppm (75 Hz, 1/2 of the FWHM of ^13^C line). The thresholds of “significant ^13^C chemical shift deviation” for individual residues were determined by the number of ^13^C nuclei that were considered. The analysis of ^13^C chemical shift deviations (relative to fibrils grown from the MM1 model membrane) was plotted in Figure 5.

The residue-specific ^13^C chemical shifts for MM2 and BM2 fibrils showed minimal deviations from the MM1 fibrils in all selectively labeled residues, including the dynamic N-terminal residue G9, suggesting that these fibrils shared common structures. On the contrary, all membrane-associated fibrils showed apparent chemical shift deviations compared to the Aβ_40_ fibrils grown in aqueous solutions (e.g., Mref1). In addition, the chemical shifts also showed obvious deviations from those reported for the first AD-patient-brain-derived Aβ_40_ fibrils (i.e., Mref2), which was derived from a patient who was diagonalized for both AD and Parkinson’s disease. However, interestingly, the ^13^C chemical shifts for the membrane-associated Aβ_40_ fibrils in the present work look similar to the proposed “common” brain-seeded Aβ_40_ fibril (i.e., Mref3) [8]. The comparison of chemical shifts indicated that the presence of membranes might lead to the formation of a predominant fibril structure independent of the detailed membrane compositions. This may explain the presence of a predominant Aβ_40_ fibril structure in AD patients because membranes are biologically relevant environments in the production and aggregation of Aβ peptides.

**Figure 6:**
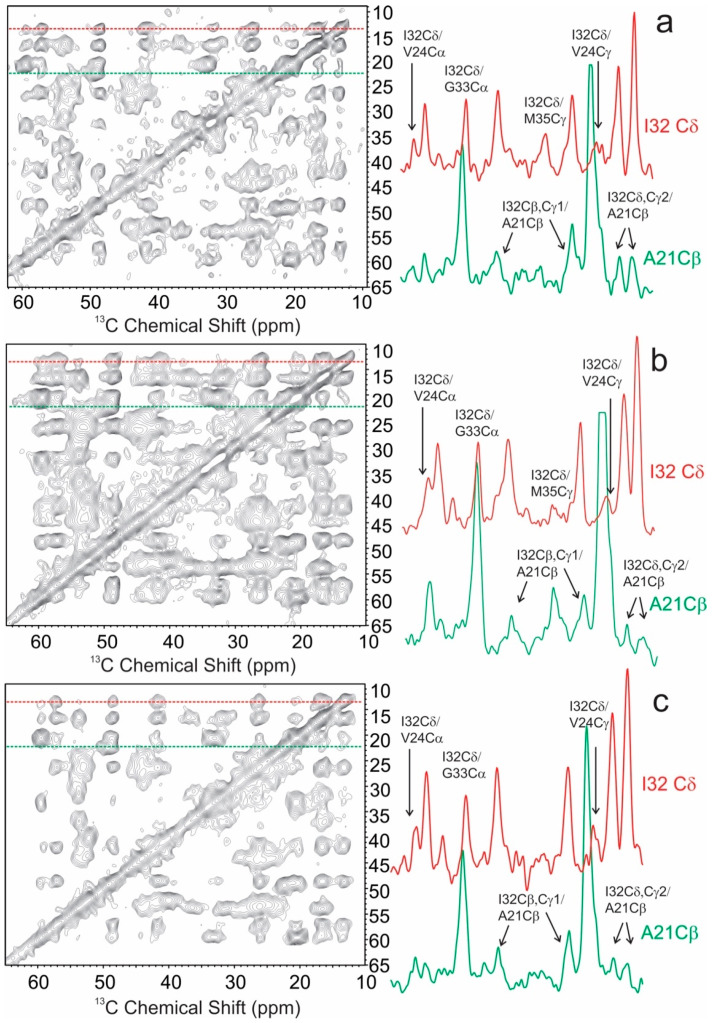
Representative long-mixing (500 ms) 2D ^13^C–^13^C spin diffusion spectra for Aβ_40_ fibrils (**a**) MM1, (**b**) MM2 and (**c**) BM2 membrane models. The same labeled samples were utilized as in Figure 4. The 1D slices highlight the inter-residue cross peaks between I32 and V24, G33, and M35, as well as between A21 and I32 in all samples.

The membrane associated Aβ_40_ fibrils also showed distinct tertiary structural features compared to those grown in aqueous and/or ex vivo conditions. The 2D spin diffusion spectra with 500 ms mixing time observed characteristic inter-residue cross peaks for residue pairs F19/L34 and V24/I32 (Figure 6) in the three membrane-associated fibrils. The ^13^C chemical shifts of all these residues showed typical β strand secondary structures (Table 3). The F19/L34 cross peaks were reported in several previously solved Aβ_40_ fibril structures [5]. However, the V24/I32 cross peaks have been rarely seen in Aβ_40_ fibrils, where residues I32 and L34 are typically within an uninterrupted β strand and V24 is usually in a loop connecting two β strand segments. In addition, the inter-residue cross peaks between A21 and I32 were also observed. These inter-residue contacts suggest a fibril core segment presumably extended from F19 to L34. Interestingly, for the two fibrils grown from model bilayers (MM1 and MM2), the inter-residue contacts were observed between I32 and M35, which was not likely to exist for the uninterrupted β strand from I31 to V39 in many solved fibril structures [4,5,37]. One previous ssNMR work on the Aβ_40_ fibril structure formed with PC/PG vesicles showed a kink at C-terminal segment from I31 to M35. It was proposed that such a kink was caused by initial interactions between Aβ and lipid bilayer during the membrane-associated nucleation step [24]. Similar kinks might be present in the current membrane-associated fibril structure.

### 3.3. The Membrane-Associated Aβ_40_ Nucleation and Fibrillization Processes Induce Rapid Cellular Membrane Leakage

**Figure 7:**
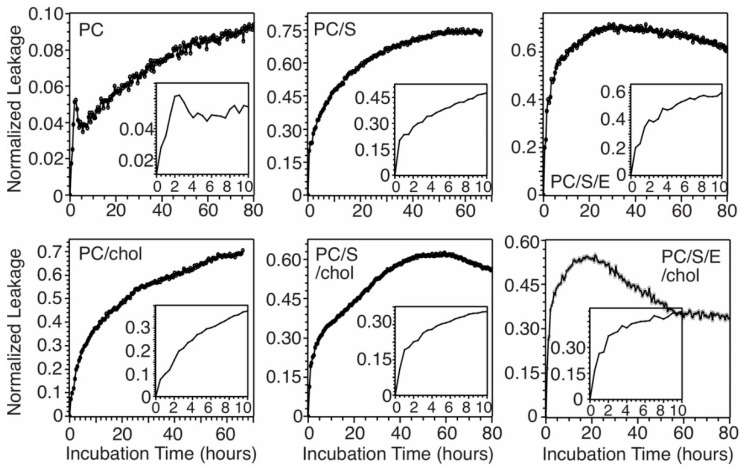
Calcein leakage assay for measuring the membrane content leakage with Aβ_40_ fibrillization in different membrane models. Insets show the rapid increase of calcein leakages within the first 10 h of incubation.

**Figure 8:**
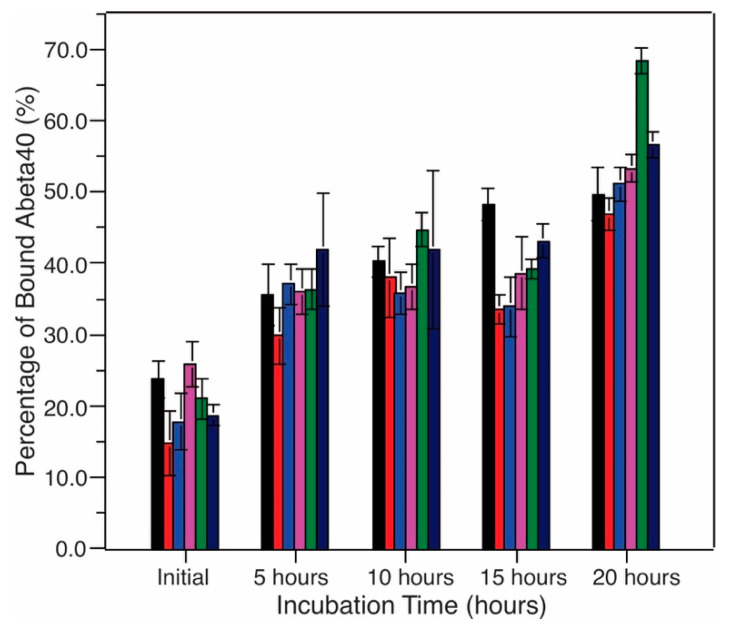
Quantitative analysis of membrane-bound Aβ_40_ as a function of incubation time within the first 20 h. Different color-codings were utilized for different model membranes: black, PC; red, PC/chol; blue, PC/S; purple, PC/S/chol; green, PC/S/E; dark navy, PC/S/E/chol. Error bars represent the s.t.d. from three independent measurements.

Disruption of cellular membranes induced by the fibrillization process has been considered as a viable cellular toxicity mechanism for Aβ peptides [2,13]. Our previous works showed that membrane content leakage served as a predominant membrane disruption pathway when Aβ_40_ formed membrane-associated fibrils [16,17,18,20,21]. The calcein leakage assays have been utilized widely to probe the membrane permeabilities of various types of Aβ aggregates, including fibrils and oligomers [39,40]. Here, we monitored the calcein leakages in a variety of model membrane systems without TG (Figure 7). Compared to the kinetics of fibrillization, which showed ~20–30-h lag periods for these bilayer models, the content leakage occurred much more rapidly and immediately after the mixing of peptides and lipid vesicles. In all systems, the membrane leakages reached at least 50% of the maximum levels (reached plateau in some cases) within the first 20 h. The comparison between ThT and membrane content leakage assays indicated that the membrane disruption occurred much earlier compared to the fibrillization process and probably through nucleation stages. It is worth noting that in all systems, there were rapid increments of membrane leakage within the first few hours (insets in Figure 7). This observation was consistent with the previous report that the initial binding between Aβ and membranes led to significant disruption [41]. With longer incubation times, the increases of membrane leakage were either decelerated or eliminated, while the ThT fluorescence emission reached the plateau. We showed previously that our sample preparation protocols led to initial a-helical membrane-bound Aβ_40_, which were not observed in the literature. The time-dependent membrane absorption of Aβ_40_ within the first 20 h of incubation was assessed using bicinchoninic acid (BCA) assay (Figure 8). The results showed that in all model systems, 15%–20% Aβ_40_ peptides were adsorbed to bilayers immediately. This population of peptides may induce the initial rapid membrane leakages, which was observed in Figure 7. Within the first 20 h (the lag periods determined in Figure 2), 50%–60% of peptides were bound to membranes, which were involved in the nucleation step and further rapid membrane disruptions. The levels of leakage seemed to be dependent on specific membrane compositions. In the model systems without cholesterol, the existence of negatively charged PS significantly enhanced the leakage (i.e., from 10% to ~60%). The presence of cholesterol, on the other hand, seemed to eliminate the deviations in leakage levels caused by individual phospholipid compositions, presumably by increasing the overall rigidity of bilayers [42].

**Figure 9:**
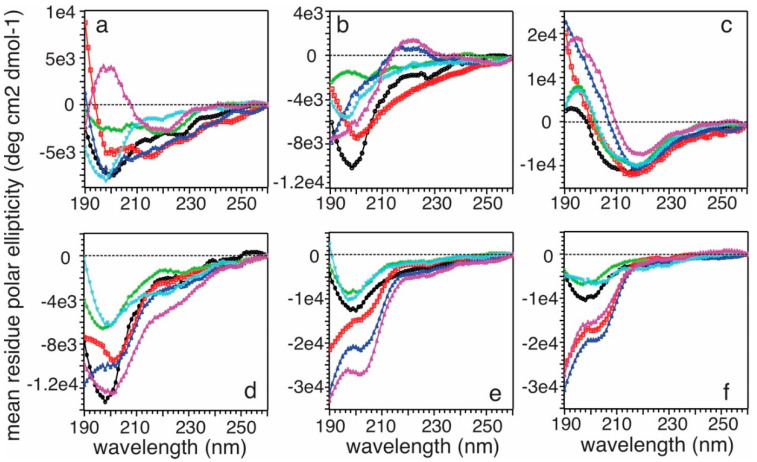
CD spectra for total and free Aβ_40_ peptides with different incubation times: (**a**,**d**) 0 h, (**b**,**e**) 5 h, (**c**,**f**) 10 h. Different color-codings were utilized for different model membranes: black, PC; red, PC/chol; blue, PC/S; purple, PC/S/chol; green, PC/S/E; dark navy, PC/S/E/chol.

Finally, circular dichroism (CD) spectroscopy was employed to study the time-dependent conformational changes of the total (both membrane-bound and nonmembrane-bound) and free (nonmembrane-bound) Aβ_40_ peptides in the time course. As shown in Figure 9, the total Aβ_40_ (top rows) adopted apparent conformational changes within the first few hours of incubation. The initial conformation was partially folded with certain negative CD signals between 210 and 230 nm and a relatively more positive signal below 200 nm. This was consistent with our previous observations of partial helical conformation for Aβ_40_ upon membrane binding [21]. The conformation became more unstructured within the first few hours (Figure 9b), but more β-strand-like with further incubation, indicated by negative CD signals between 210 and 220 nm (Figure 9c). On the contrary, the free Aβ_40_ left in solution did not show conformational changes within the same time course and remained mostly unstructured. The CD data indicated that only the membrane bound Aβ_40_ had structural changes at the early stage of aggregation (i.e., <10 h) and the Aβ_40_ nucleation process mainly occurred with the association of membranes rather than in aqueous solutions.

## 4. Discussion

The biological implications of this work are two-fold. First of all, it provides the time scales of membrane-associated fibrillization process relative to the membrane disruption, which facilitates future studies on the crucial intermediate states that are responsible for such membrane disruption and the molecular mechanisms. Fibrillization of Aβ is considered as a pathologically relevant process in AD. When the process occurs with the presence of membranes, which represent biologically relevant environments in human brains, there may be multiple pathways that lead to various types of end products [20]. Our previous studies showed that Aβ mainly form fibrils in the presence of phospholipid bilayer models when the peptide concentrations were relatively low (i.e., less than 50 mM) and the peptide-to-lipid molar ratios were relatively high (i.e., ~1:30) [20] and induced membrane content leakage along with the fibrillization processes [18]. In addition, the presence of membranes promoted an initial Aβ_40_ peptide conformational change upon the first few hours of incubation from partial a-helical to random coil, prior to the presence of typical β-strands for high-order amyloid aggregates [21]. The outcomes of the present work, together with our previous conclusions, raise a uniform hypothesis for the membrane-associated Aβ_40_ fibrillization process in multiple types of bilayer model systems: Within the first few hours’ (e.g., 5 h) incubation, around 30%–40% peptides were rapidly adsorbed to membranes (Figure 8), which adopted a transient helical conformation initially and converted to random coils [21]. Within the next 20 h, which is the nucleation step with no ThT fluorescence emission increments (Figure 2), an additional 15%–20% Aβ_40_ are adsorbed. These initial membrane binding and nucleation steps cause rapid membrane content leakage (Figure 7), presumably because of the dramatic structural changes in the membrane-bound domains of peptides. At the end of nucleation steps, the peptides show a large abundance of β-strand conformation [16]. Further incubation causes elongation of fibrils based on the existing nucleus formed with the initially membrane-bound peptides, and this process induces modest membrane leakages in some model bilayers (Figure 7). Therefore, future studies on the membrane-disrupting intermediate states of Aβ_40_ peptides will focus on the first ~20 h of incubation.

A second implication is that the high-resolution ssNMR studies on the molecular structures of membrane-associated Aβ_40_ fibrils showed that the presence of membranes reduces the heterogeneity of fibrils, especially in the rigid core region, because of the similarity in chemical shifts found among fibrils grown from various membrane systems. Interestingly, fibrils grown from membranes seem to have a relatively smaller core compared to nonmembrane-associated fibrils [5,37,43]. The fibril core is likely to start after residue L17 in the present work, while in the reported fibrils from aqueous solutions, the fibril cores extend towards K16, or in some cases, residue G9 [38]. Contacts between residues V24 and I32 found in the membrane-associated fibrils suggest different core structures from the aqueous fibrils. The ^13^C chemical shift features of the three membrane-associated fibrils seem to be similar to the reported predominant Aβ_40_ fibril grown from AD human-brain-extracted seeds [7,8], meaning that membranes provide biologically relevant environments for the studies of fibril molecular structures.

Although the presence of membranes may lead to a uniform Aβ_40_ fibrillization process with reduced heterogeneity in the resultant fibrils, the detailed physicochemical properties of fibril formation and influences on membranes, such as the fibrillization kinetics and membrane leakage levels, are sensitive to membrane compositions. The presence of heterogeneous membrane compositions, their asymmetric distributions across the two leaflets, and their time-dependent changes along the aging process are critical factors in the membrane-related Aβ neuronal toxicity mechanisms [12,44,45,46,47]. Analysis of the molecular compositions in rSPMV showed that in addition to the phospholipids, cholesterol, and triglycerides are essential components in biological membranes. Our results imply that the presence of triglycerides significantly decelerates the fibrillization process in all tested model bilayers with various phospholipids/cholesterol compositions. It is reported that triglycerides are essential components in lipid rafts, which are subdomains in lipid bilayers with relatively lower densities [26]. It is also suggested that the lipid rafts served as possible pools to concentrate Aβ peptides inside of cellular plasma membranes. Therefore, the triglycerides in lipid rafts might keep the highly concentrated Aβ from aggregation in cellular plasma membranes. Cholesterol shows significant influences on both the membrane-associated fibrillization kinetics and the fibrillization-induced membrane content leakage levels. Our results suggest that cholesterol may serve as regulation factors in biological membranes to modulate the Aβ aggregation process. The mammalian cellular plasma membranes possess asymmetric phospholipid distribution in their outer and inner leaflets [33]. Typically, the outer leaflet is rich in neutral PC and the inner leaflet contains more PS and PE. In the meanwhile, the distribution of cholesterol was uneven with more populations in the inner leaflets. It was shown that cholesterol might migrate from the inner to the outer leaflet with aging [48]. Thus, it is possible that the extracellular Aβ fibrillization induces less membrane content leakage at younger ages because the outer leaflet contains mostly PC. However, the fibrillization-induced membrane content levels may increase with aging because of the addition of cholesterol to the outer leaflet. For the inner leaflet, however, the extent of membrane leakage may be lower at the younger ages because our data showed that the combination of PS, PE, and cholesterol led to bilayers with lower leakage levels compared to those with only PS and PE. Overall, both leaflets might become more permeable with the aging-dependent migration of cholesterol.

In summary, we showed in the present work that when Aβ_40_ was incubated with model membranes at relatively low concentrations (i.e., 10 mM) and high peptide-to-lipid molar ratio (i.e., 1:30), it adopted a uniform membrane-associated nucleation process that led to membrane leakages and a predominant fibril structure. Future characterizations of both the membrane-associated fibril structure and the early-stage membrane-active intermediate states will help to understand the molecular basis of cellular membrane disruptions induced by Aβ_40_ peptides.

## Figures and Tables

**Table 1:** List of model bilayers used in the present work.

Lipid Compositions	Molar Ratio	Lipid Compositions	Molar Ratio
PC ^1^ (MM1)	---	PC/PG/chol/SM/GM ^2^	1.00/1.00/1.33/1.00/0.10
PC/chol	4.68/1.73	PC/chol/TG	4.68/1.73/0.05
PC/PS/chol	4.68/1.00/1.73	PC/PS/chol/TG	4.68/1.00/1.73/0.05
PC/PS/PE/chol	4.68/1.00/1.00/1.73	PC/PS/PE/chol/TG	4.68/1.00/1.00/1.73/0.05
PC/TG	4.68/0.05	PC/PS/TG	4.68/1.00/0.05
PC/PS/PE/TG	4.68/1.00/1.00/0.05	PC/PS	4.68/1.00
PC/PS/PE	4.68/1.00/1.00		

^1^ The abbreviations were used: PC, phosphatidylcholine; PG, phosphatidylglycerol; chol, cholesterol; SM, sphingomyelin; GM, ganglioside GM1; PS, phosphatidylserine; PE, phosphatidylethanolamine; TG, triglycerides. ^2^ This membrane composition was used in our previous studies.

**Table 2:** Summary of the best-fit parameters for ThT kinetics.

Membrane Composition ^1^	A0 (a.u.) ^2^	t_lag_ (hours)	*k* (hour^−1^)
PC	1508 (143) ^3^	19.21 (1.64)	0.237 (0.036)
PC/chol	1636 (171)	17.09 (5.32)	0.183 (0.067)
PC/PS	1699 (97)	26.89 (1.75)	0.186 (0.025)
PC/PS/chol	1683 (129)	20.46 (1.78)	0.283 (0.048)
PC/PS/PE	1762 (114)	21.43 (3.20)	0.225 (0.038)
PC/PS/PE/chol	2057 (125)	11.84 (4.39)	0.200 (0.048)
PC/TG	498 (103)	51.87 (8.36)	0.099 (0.004)
PC/chol/TG	388 (63)	11.59 (3.82)	0.110 (0.012)
PC/PS/TG	487 (114)	51.55 (7.06)	0.041 (0.010)
PC/PS/chol/TG	401 (41)	23.16 (1.14)	0.068 (0.004)
PC/PS/PE/TG	481 (118)	52.33 (6.74)	0.041 (0.010)
PC/PS/PE/chol/TG	446 (29)	25.74 (1.95)	0.049 (0.002)
BM1	1184 (93)	48.00 (7.31)	0.059 (0.005)
BM2	1262 (149)	32.60 (5.44)	0.052 (0.006)
BM3	1382 (44)	55.81 (7.50)	0.075 (0.022)

^1^ Detailed molar ratio described in Table 1; ^2^ Definitions of fitting parameters provided in Section 2.6; ^3^ Errors from three independent measurements in parentheses.

**Table 3:** Residue-specific ^13^C chemical shifts for membrane-associated Aβ_40_ fibrils.

**MM1**					
	C	Ca	Cβ	Cg	Cd
G9	171.1	44.9			
L17	174.0	53.3	43.9	27.0, 24.9	
F19	174.3	56.8	42.4		
A21	174.3	49.3	22.4		
V24	176.3	59.3	32.6	20.8	
N27	173.5	52.4	40.5	175.8	
I32	175.7	57.0	42.0	26.3, 16.5	13.5
G33	171.6	48.5			
L34	174.8	54.3	46.8	28.5, 26.4	
M35	173.2	53.6	36.5	31.5	16.4
**MM2**					
	C	Ca	Cβ	Cg	Cd
G9	170.7	44.8			
L17	174.2	53.1	44.2	27.1, 24.3	
F19	174.3	56.7	42.0		
A21	174.6	49.2	22.4		
V24	175.7	59.5	33.2	20.5	
N27	173.2	52.3	39.9	175.7	
I32	175.7	57.3	41.8	26.3, 16.7	13.3
G33	171.7	48.6			
L34	174.9	54.3	46.7	28.5, 26.1	
M35	173.3	53.8	36.5	31.8	16.5
**BM2**					
	C	Ca	Cβ	Cg	Cd
G9	170.5	44.8			
L17	173.9	53.0	44.3	27.0, 24.3	
F19	174.0	56.4	41.8		
A21	174.3	49.5	22.4		
V24	176.1	59.5	32.7	20.7	
N27	173.0	52.4	39.9	175.8	
I32	175.7	57.0	42.0	26.2, 16.5	13.6
G33	171.5	48.5			
L34	174.6	54.0	46.5	28.2, 25.7	
M35	173.2	53.9	36.8	32.1	17.1

## References

[1] Hardy J.A., Higgins G.A. (1992). Alzheimer’s disease: The amyloid cascade hypothesis. Science.

[2] Karran E., Mercken M., De Strooper B. (2011). The amyloid cascade hypothesis for Alzheimer’s disease: An appraisal for the development of therapeutics. Nat. Rev..

[3] Reitz C. (2012). Alzheimer’s Disease and the amyloid cascade hypthesis: A critical review. Int. J. Alzheimers Dis..

[4] Tycko R. (2011). Solid-state NMR studies of amyloid fibril structure. Annu. Rev. Phys. Chem..

[5] Tycko R. (2015). Amyloid polymorphism: Structural basis and neurobiological relevance. Neuron.

[6] Lu J.X., Qiang W., Yau W.M., Schwieters C.D., Meredith S.C., Tycko R. (2013). Molecular structure of β-amyloid fibrils in Alzheimer’s disease brain tissue. Cell.

[7] Qiang W., Yau W.M., Lu J.X., Collinge J., Tycko R. (2017). Structural variation in amyloid-beta fibrils from Alzheimer’s disease clinical subtypes. Nature.

[8] Ghosh U., Yau W.M., Tycko R. (2018). Coexisting order and disorder within a common 40-residue amyloid-beta fibril structure in Alzheimer’s disease brain tissue. Chem. Commum..

[9] Thinakaran G., Koo E.H. (2008). Amyloid precursor protein trafficking, processing and function. J. Biol. Chem..

[10] O’Brien R.J., Wong P.C. (2011). Amyloid precursor protein processing and Alzheimer’s disease. Annu. Rev. Neurosci..

[11] Kawarabayashi T., Shoji M., Younkin L.H., Lin W., Dickson D.W., Murakami T., Matsubara E., Abe K., Ashe K.H., Younkin S.G. (2004). Dimeric amyloid beta peotein rapidly accumulates in lipid rafts followed by Apolipoprotein E and phosphorlated Tau accumulation in the Tg2576 mouse model of Alzheimer’s disease. J. Neurosci..

[12] Eckert G.P., Wood W.G., Mueller W.E. (2010). Lipid membranes and beta-amyloid: A harmful connection. Curr. Protein Pep. Sci..

[13] Butterfield S.M., Lashuel H.A. (2010). Amyloidogenic protein-membrane interactions: Mechanistic insight from model systems. Angew. Chem..

[14] Sciacca M.F.M., Brender J.R., Lee D.K., Ramamoorthy A. (2012). Phosphatidylethanolamine enhances amyloid fiber dependent membrane fragmentation. Biochemistry.

[15] Vestergaard M.C., Morita M., Hamada T., Takagi M. (2013). Membrane fusion and vesicular transformation induced by Alzheimer’s amyloid beta. Biochim. Biophys. Acta.

[16] Qiang W., Akinlolu R.D., Nam M., Shu N. (2014). Strcutral evolution and membrane interaction of the 40-residue beta amyloid peptides: Differences in the initial proximity between peptides and the membrane bilayer studied by solid-state nulear magnetic resonance spectroscopy. Biochemistry.

[17] Qiang W., Yau W.M., Schulte J. (2015). Fibrillation of beta amyloid peptides in the presence of phospholipid bilayers and the consequent membrane disruption. Biochim. Biophys. Acta.

[18] Akinlolu R.D., Nam M., Qiang W. (2015). Competition between fibrillation and induction of vesicle fusion for the membrane-associated 40-residue β-amyloid peptides. Biochemistry.

[19] Tofoleanu F., Brooks B.R., Buchete N.V. (2015). Modulation of Alzheimer’s Abeta protofilament-membrane interactions by lipid headgroups. ACS Chem. Neurosci..

[20] Delgado D.A., Doherty K., Cheng Q., Kim H., Xu D., Dong H., Grewer C., Qiang W. (2016). Distinct membrane disruption pathways induced by the 40-residue β-amyloid peptides. J. Biol. Chem..

[21] Cheng Q., Hu Z.W., Doherty K.E., Tobin-Miyaji Y.J., Qiang W. (2018). The on-fibrillation-pathway membrane content leakage and off-fibrillation-pathway lipid mixing induced by 40-residue β-amyloid peptides in biologically relevant model liposomes. Biochim. Biophys. Acta.

[22] Murphy R.M. (2007). Kinetics of amyloid formation and membrane interaction with amyloidogenic proteins. Biochim. Biophys. Acta.

[23] Yu X., Wang Q.M., Pan Q.F., Zhou F.M., Zheng J. (2013). Molecular interactions of Alzheimer amyloid-beta oligomers with neutral and negatively charged lipid bilayers. Phys. Chem. Chem. Phys..

[24] Niu Z., Zhao W., Zhang Z., Xiao F., Tang X., Yang J. (2014). The molecular structure of Alzheimer β-amyloid fibrils formed in the presence of phospholipid vesicles. Angew. Chem. Int. Ed..

[25] Bermejo M.K., Milenkovic M., Salahpour A., Ramsey A.J. (2014). Preparation of synaptic plasma membrane and postsynaptic density proteins using a discontinuous sucrose gradient. J. Vis. Exp..

[26] Brogden G., Propsting M., Adamek M., Naim H.Y., Steinhagen D. (2014). Isolation and analysis of membrane lipids and lipid rafts in common carp (*Cyprinus carpio* L.). Comp. Biochem. Physiol..

[27] Fuchs B., Suss R., Teuber K., Eibisch M., Schiller J. (2011). Lipid analysis by thin-layer chromatography—A ewview of the current state. J. Cheromathgr. A.

[28] van Meer G., de Kroon A.I.P.M. (2011). Lipid map of the mammalian cell. J. Cell Sci..

[29] Tashima Y., Oe R., Lee S., Sugihara G., Chambers E.J., Takahashi M., Yamada T. (2004). The effects of cholesterol and monosialoganglioside (GM1) on the release and aggregation of amyloid beta-peptide from liposomes prepared from brain membrane-lipid lipids. J. Biol. Chem..

[30] Chi E.Y., Frey S.L., Lee K.Y.C. (2007). Ganglioside GM1-mediated amyloid-beta fibrillogenesis and membrane disruption. Biochemistry.

[31] Hellstrand E., Sparr E., Linse S. (2010). Retardation of Abeta fibril formation by phospholipid vesicles depends on membrane phase behavior. Biophys. J..

[32] Fonsece A.C., Resende R., Oliveira C.R., Pereira C.M.F. (2010). Cholesterol and statins in Alzheimer’s disease: Current controversies. Exp. Neurol..

[33] Wood W.G., Igbavboa U., Muller W.E., Eckert G.P. (2011). Cholesterol asymmetry in synaptic plasma membranes. J. Neurochem..

[34] Petkova A.T., Yau W.M., Tycko R. (2006). Experimental constraints on quaternary structure in Alzheimer’s beta-amyloid fibrils. Biochemistry.

[35] Paravastu A.K., Leapman R.D., Yau W.M., Tycko R. (2008). Molecular structural basis for polymorphism in Alzheimer’s beta-amyloid fibrils. Proc. Natl. Acad. Sci. USA.

[36] Qiang W., Yau W.M., Luo Y.Q., Mattson M.P., Tycko R. (2012). Antiparallel beta-sheet architecture in Iowa-mutant beta-amyloid fibrils. Proc. Natl. Acad. Sci. USA.

[37] Sgourakis N.G., Yau W.M., Qiang W. (2015). Modeling an in-register, parallel “iowa” Abeta fibril structure using solid-state NMR data from labeled samples with Rosetta. Structure.

[38] Hu Z.W., Vugmeyster L., Au D.F., Ostrovsky D., Sun Y., Qiang W. (2019). Molecular structure of an N-terminal phosphorylated beta-amyloid fibril. Proc. Natl. Acad. Sci. USA.

[39] Williams T.L., Serpell L.C. (2011). Membrane and surface interactions of Alzheimer’s Abeta peptide—Insights into the mechanism of cytotoxicity. FEBS J..

[40] Williams T.L., Johnson B.R.G., Urbanc B., Jenkins T.A., Connel S.D.A., Serpell L.C. (2011). Abeta42 olligomers, but not fibrilsm simultaneously bind to and cause damage to ganglioside-containing lipid membranes. Biochem. J..

[41] Williams T.L., Day I.J., Serpell L.C. (2010). The effect of Alzheimer’s Abeta aggregation state on the permeation of biomimetic lipid vesicles. Langmuir.

[42] Wolozin B. (2001). A fluid connection: Cholesterol and Abeta. Proc. Natl. Acad. Sci. USA.

[43] Bertini I., Grnnelli L., Luchinat C., Mao J.F., Nesi A. (2011). A new structural model for A beta(40) fibrils. J. Am. Chem. Soc..

[44] Marquardt D., Geier B., Pabst G. (2015). Asymmetric lipid membranes: Towards more realistic model systems. Membranes.

[45] Yang Y., Yao H., Hong M. (2015). Distinguishing bicontinuous lipid cubic phases from istropic membrane morphologies using 31P solid-state NMR spectroscopy. J. Phys. Chem. B.

[46] Andreasen M., Lorenzen N., Otzen D. (2015). Interactions between misfolded protein oligomers and membranes: A central topic in neurogenenerative diseases?. Biochim. Biophys. Acta.

[47] Sugiura Y., Ikeda K., Nakano M. (2015). High membrane curvature enhances binding, conformational changes, and fibrillation of amyloid-beta on lipid bilayer surface. Langmuir.

[48] Puglielli L., Tanzi R.E., Kovacs D.M. (2013). Alzheimer’s disease: The cholesterol connection. Nat. Neurosci..

